# Supplementary material for: Elicitation of potent serum neutralizing antibody responses in rabbits by immunization with an HIV-1 clade C trimeric Env derived from an Indian elite neutralizer
Source: PLoS Pathog. 2021 Apr 7;17(4):e1008977. doi: 10.1371/journal.ppat.1008977 (PMC8055034; doi:10.1371/journal.ppat.1008977)
Supplement: S1 Table — (DOCX) [file ppat.1008977.s001.docx]

**Table S1**. Mapping specificities of the autologous and heterologous plasma neutralizing antibodies obtained from the Indian elite neutralizer.

|  |  |  | **Fold reduction in ID_50_ values** | |
| --- | --- | --- | --- | --- |
| Envs | Clade | Autologous/Heterologous | 92BR020  (N332A) | 92BR020  (N301A/N332A) |
| 92BR020 | B | Heterologous | >33.15 | No change |
| 4-2. J41 | C | Heterologous | >37.65 | No change |
| Q23.17 | A | Heterologous | >23.65 | No change |
| IAVI C22 | C | Heterologous | >42.50 | No change |
| CH038 | B/C | Heterologous | >20.00 | No change |
| Q259.D22.2 | A | Heterologous | >60.5 | No change |
| JRCSF | B | Heterologous | >14.55 | No change |
| PG80v1.eJ7 | C | Autologous (wild type) | No change | No change |
| PG80v1.eJ19 | C | Autologous (wild type) | No change | No change |
| PG80v2.eJ38 | C | Autologous (wild type) | No change | No change |
| PG80v2.eJ38(v1. eJ7 V1V2) | C | Autologous (chimera) | No change | No change |
| PG80v2.eJ38(v1. eJ7 V3C3) | C | Autologous (chimera) | No change | No change |
| PG80v2.eJ38(v1. eJ7 V3C4) | C | Autologous (chimera) | No change | No change |
| PG80v2.eJ38(v1. eJ7 C4V5) | C | Autologous (chimera) | No change | No change |

*The heterologous neutralization specificity was mapped by using 92BR020 N332A and 92BR020 N301A / N332. As shown in this Table S1, plasma antibodies obtained from the elite neutralizer (32) depleted with 92BR020 N332A but not 92BR020 N301A / N332A gp120 proteins demonstrated a significant reduction of neutralization of heterologous viruses, when compared with that of undepleted plasma antibodies, which indicated that the neutralization breadth of plasma antibodies developed in this Indian elite neutralizer correlated with their target specificity to conserved V3 glycans. Note that comparable data obtained when plasma antibodies were depleted with 92BR020 gp120 with that of 92BR020 N332A (data not shown). ID_50_ values refer to the reciprocal dilution conferring 50% neutralization of Env-pseudotyped viruses in TZM-bl cells. Fold reduction in ID_50_ values refer to the ID_50_ values observed with depleted plasma antibodies compared to undepleted plasma antibodies.*
