# Supplementary figures and images for: Elicitation of potent serum neutralizing antibody responses in rabbits by immunization with an HIV-1 clade C trimeric Env derived from an Indian elite neutralizer
Source: PLoS Pathog. 2021 Apr 7;17(4):e1008977. doi: 10.1371/journal.ppat.1008977 (PMC8055034; doi:10.1371/journal.ppat.1008977)

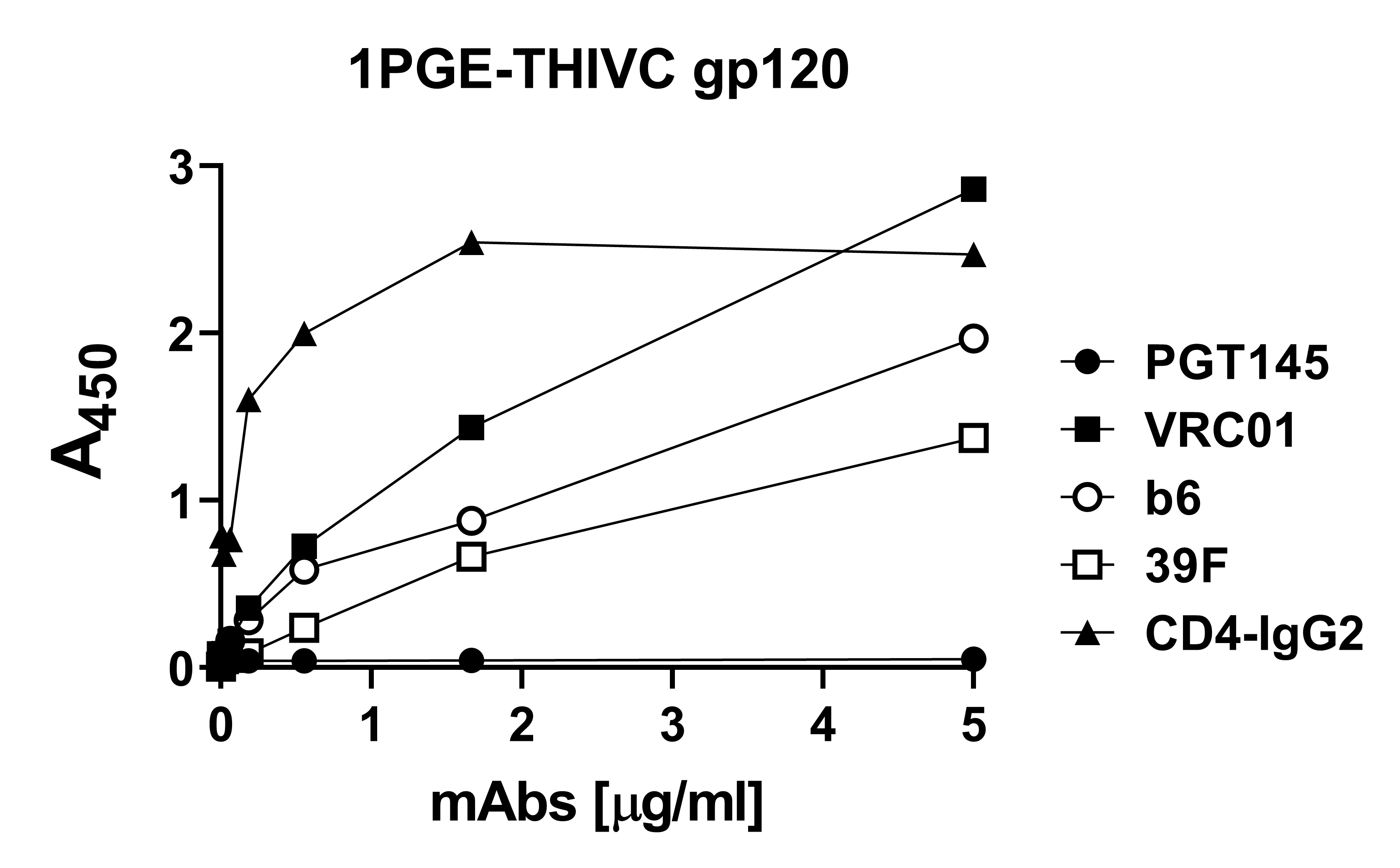

Supplement: S1 Fig — PGT145 bnAb was taken as a negative control. (TIF) [file ppat.1008977.s002.tif]

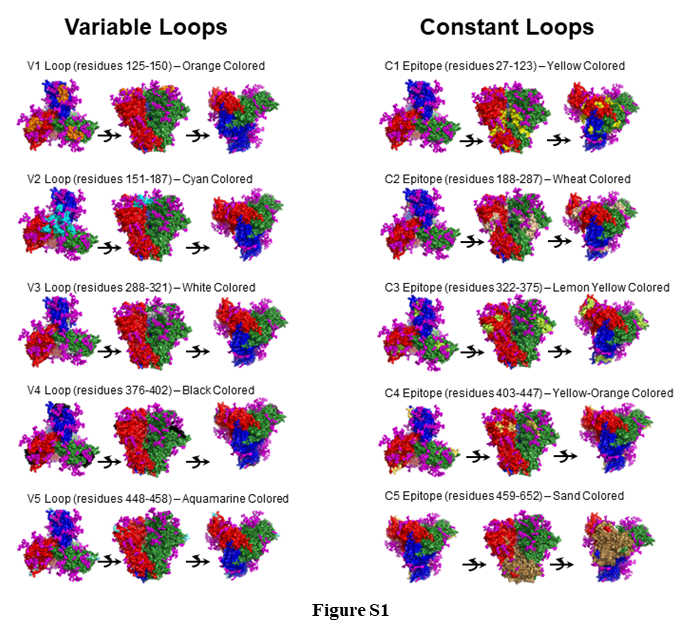

Supplement: S2 Fig — In parentheses, highlighted residues and their respective colors are mentioned. The black arrows aid in providing the rotations done in model to present the image. (TIF) [file ppat.1008977.s003.tif]

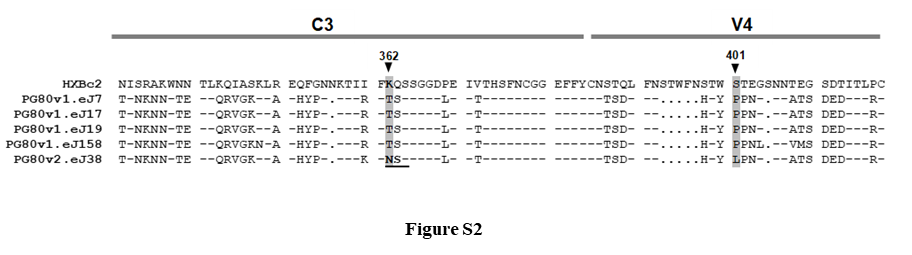

Supplement: S3 Fig — Amino acid numbering is made based on HXbc2 sequence. Amino acid residues in C3 and V4 that form key epitopes targeted by neutralizing antibodies induced in rabbits are highlighted. The glycan residue at the 362 position is underscored. (TIF) [file ppat.1008977.s004.tif]
